# A Geospatial Study of Opioid Overdose Risk in Diverse Regions

**DOI:** 10.23889/ijpds.v9i5.2700

**Published:** 2024-09-18

**Authors:** Hashan Fernando, Adam Spannaus, Anuj Kapadia

**Affiliations:** 1 Oak Ridge National Laboratory; 2 University of Tennessee

Over the past two decades fatalities from opioid overdoses (OD) in the United States have increased at an alarming rate, and there exist regional disparities in the number of opioid fatalities within the US. A comprehensive understanding of the underlying factors contributing to this geographic variation remains elusive however. In this study, we consider the Social Vulnerability Index (SVI) census variables and their influence on opioid overdose within diverse geographic regions, each exhibiting different socio-economic factors. To investigate these disparate outcomes, we use Social Vulnerability Index data from the year 2018 alongside mortality data obtained from the CDC WONDER database.

The factors that contribute to opioid OD fatalities can vary from one region to another due to differences in socio-economic characteristics. To investigate these regional differences, we have chosen a set of variables for each region which are relevant to understanding the opioid OD fatalities in three different geographical regions: the Washington D.C metropolitan area, Wyoming, Tennessee. The impact of each factor on the epidemic's propagation varies between these regions however. To identify patterns in socioeconomic factors driving the opioid crisis, we applied methods from topological data analysis to identify potential correlations between social vulnerability factors and the distribution of drug overdose fatalities. In conclusion, our work highlights the importance of the socio-economic factors in understanding the dynamics of the opioid epidemic.

